# Still not over: Africa CDC and WHO issue third reaffirmation of mpox emergency

**DOI:** 10.4102/jphia.v16i1.1556

**Published:** 2025-08-20

**Authors:** Ngashi Ngongo, Yap Boum, Kyeng Mercy, Mosoka P. Fallah, Nebiyu Dereje, Michel Muteba, Jean Kaseya

**Affiliations:** 1 Africa Centres for Disease Control and Prevention, Addis Ababa, Ethiopia; 2 World Health Organization, Brazzaville, Democratic Republic of the Congo

## Introduction

Mpox, a zoonotic infection caused by an orthopoxvirus and historically endemic to several African countries, has re-emerged as a persistent and complex public health threat.^1^ Despite the initial global focus during the 2022 multi-country outbreaks, the burden of disease has remained largely concentrated in Africa, where transmission continues and health systems face ongoing challenges.^2^ In response to sustained human-to-human transmission and evolving epidemiological dynamics, the Africa Centres for Disease Control and Prevention (Africa CDC) and the World Health Organization Regional Office for Africa (WHO AFRO) jointly declared mpox a public health emergency of continental security and of international concern in August 2024.^3,4,5^ This designation was formally extended in February 2025, and once again in June 2025, following continued spread and the emergence of new transmission hotspots. The decision is grounded in robust epidemiological evidence and reflects a commitment to ensuring equity in global health preparedness and response.

## Current epidemiological trends

Between January 2024 and epidemiological week 23 of 2025, the African continent reported a cumulative total of 148 308 suspected and 40 674 confirmed mpox cases, including 1816 deaths among suspected cases (case fatality rate [CFR]: 1.2%) and 200 deaths among confirmed cases (CFR: 0.5%). These cases were reported across 26 of the 55 African Union (AU) Member States. By 18 June 2025, the number of confirmed cases in 2025 (20 936) had already surpassed the total recorded in 2024 (19 738) by 6%, with active outbreaks continuing in 19 countries.

Temporal trends show a steady increase in the outbreak. Comparing the last four months of 2024 (September–December) with the first four months of 2025 (January–April), there is a rise in the weekly average of suspected cases (from 2777 to 2874) and deaths (from 488 to 672). A more detailed comparison of the 3-week periods before each of the three public health emergency declarations reveals a consistent increase in both suspected cases – from 1468 before the first declaration to 2838 and 3430 before the second and third – and reported deaths, which went up from 229 to 689 and 845, respectively.

In the first quarter of 2025, four additional AU Member States – Sierra Leone, Malawi, South Sudan, and Tanzania – reported imported cases. Concurrently, three countries outside the African continent also confirmed mpox cases, underlining the persistent risk of international spread.

While overall case counts have increased, recent data suggest evolving dynamics in transmission. A decline in reported cases and deaths has been observed over the past 2 months in the Democratic Republic of Congo (DRC), Uganda, and Burundi – countries that together accounted for over 85% of all cases as of February 2025. However, this trend warrants cautious interpretation. Surveillance in several countries remains largely passive, and testing coverage – particularly in the DRC – remains suboptimal. Nonetheless, targeted interventions, including vaccination of high-risk groups and infection prevention and control (IPC) measures in identified hotspots, may be contributing to the observed decline.

In contrast, Sierra Leone has emerged as a new epicentre. The country, which had previously reported fewer than 10 confirmed cases per week in January 2025, accounted for over 50% of all confirmed cases between epidemiological weeks 19 and 22, with weekly totals exceeding 400 confirmed cases during that period.

In addition, laboratory surveillance capacity continues to present challenges. In 2025 (epidemiological weeks 1–23), testing coverage was 55%, a slight decline from 57% in 2024. More concerning is the marked increase in the test positivity rate (TPR), which rose from 38.8% in 2024 to 55% by week 23 of 2025. This high TPR suggests persistent community transmission, delayed care seeking, and potential under-detection of cases.

These findings underscore the urgent need to strengthen active surveillance, expand diagnostic testing, improve IPC measures, and scale up targeted vaccination to curb the evolving outbreak.

## Rationale for emergency extension

The joint decision is based on the following criteria:

### Transmission dynamics

In 2025, mpox demonstrated sustained human-to-human transmission in at least 21 countries, marking a significant expansion of its geographic footprint. Notably, five new African countries – Ethiopia, Malawi, Sierra Leone, South Sudan, and Tanzania – reported indigenous transmission for the first time, highlighting the virus’s continued regional spread. In parallel, three non-African countries – China, the United Arab Emirates, and Switzerland – also documented their first-ever mpox cases, signalling the virus’s growing international reach.^6,7^ In countries such as Sierra Leone and Uganda, initial cases attributed to importation have evolved into sustained community transmission, raising concern over silent and undetected chains of infection. Genomic analyses have linked the outbreaks in non-African countries to Clade Ib, currently circulating in the DRC and parts of East Africa, further underscoring the role of regional transmission dynamics in cross-border spread.^7^ These patterns point to both insufficient containment and the increased transmissibility of circulating lineages, reaffirming the need for enhanced surveillance, contact tracing, and coordinated response mechanisms.

### Disease burden and severity

The burden of mpox in Africa has continued to escalate in 2025, with a notable increase in both suspected and confirmed cases across the continent. Between January 2025 and May 2025, a total of 67 510 suspected cases were reported, including 20 066 confirmed cases – figures that already represent 87% of all suspected cases and exceed 100% of confirmed cases reported throughout the entire year of 2024. This trend underscores a sharp rise in transmission and case detection. A comparison of weekly averages further confirms this upward trajectory: between September and December 2024, countries reported an average of 2777 suspected cases and 488 confirmed cases per week, which rose to 2874 suspected and 682 confirmed cases per week from January 2025 to April 2025. Despite the growing caseload, the CFR has slightly improved, declining from 1.3% to 1.0% among suspected cases, and from 0.8% to 0.5% among confirmed cases over the same period. This may reflect increased care seeking with reporting of mild cases and/or improved clinical management. However, emerging hotspots demand urgent attention: during epidemiological week 22, Sierra Leone – a newly affected country – accounted for 63% of all confirmed cases continent-wide, highlighting both the expanding geographic footprint of the outbreak and the need for intensified response in newly affected areas.

### Clade

The mpox epidemic in Africa is marked by a complex and evolving clade landscape, with multiple concurrent outbreaks driven by genetically distinct viral lineages. Currently, Clade Ia is circulating predominantly in Central Africa, including the Central African Republic, Republic of Congo, and the DRC. In contrast, Clade Ib – a genetically distinct and increasingly widespread lineage – is affecting Central, East, and Southern Africa, with confirmed transmission in countries such as DRC, Uganda, Burundi, Rwanda, Kenya, Tanzania, Ethiopia, Malawi, Zambia, Zimbabwe, and South Africa. Meanwhile, Clade IIa is primarily reported in West African nations such as Côte d’Ivoire, Liberia, Nigeria, and Sierra Leone, while Clade IIb has been identified in both West and North Africa, including Nigeria, Liberia, Sierra Leone, and Morocco. Of growing concern is the emergence of novel mutations, such as lineage G.1, currently predominant in Sierra Leone, which is phylogenetically related to Clade IIb in Nigeria.^7,8^ These newly emerging lineages underscore the need for enhanced genomic surveillance and targeted research to better understand their transmission dynamics, virulence profiles, and potential implications for control strategies. The geographic and genetic diversity of circulating mpox clades further reinforces the necessity of adopting regionally tailored responses rather than a uniform continental approach.

### Risk of international spread

The risk of international spread of mpox remains high across West, Central, and East Africa, driven by a combination of epidemiological, geopolitical, and structural factors. In particular, the DRC continues to experience rising transmission amid ongoing armed conflicts, despite some localised improvements in security. These conflicts have triggered widespread population displacements, further straining health systems and disrupting surveillance and containment efforts. The situation is compounded by high levels of population mobility and intensifying cross-border movements, which enable the virus to spread silently into neighbouring countries and beyond. Moreover, the forces of globalisation – including the expansion of international trade and travel networks – and the resurgence of air traffic following pandemic-related declines have significantly increased the risk of transcontinental transmission. Recent detections of mpox in non-endemic regions such as Asia, Europe and Middle East underscore the urgency of strengthened international coordination, cross-border surveillance, and the integration of mpox response into broader global health security frameworks.

### Inadequate response capacity

The establishment of the continental Incident Management Support Team (IMST) has marked a paradigm shift in Africa’s outbreak response architecture. However, the global response to mpox remains markedly insufficient. A combination of chronically weak health systems, constrained access to vaccines and other medical countermeasures and declining international health financing – particularly following aid reductions by the United States (US), United Kingdom (UK), Italy, and others – has severely undermined containment efforts. During the second phase of the mpox response (March 2025–August 2025), only 500 000 vaccine doses were secured against an estimated need of 6.4 million, with minimal funding available for IPC commodities. Many affected countries in Africa face chronic underinvestment in public health infrastructure, resulting in limited capacity for disease surveillance, laboratory diagnosis, case management, and outbreak containment. Despite the clear signals of sustained transmission and emerging viral diversity, global interest in mpox and funding has waned, relegating mpox to the margins of pandemic preparedness agendas. This lack of sustained political and financial commitment has hampered the development of context-appropriate response strategies and slowed efforts to scale up regional manufacturing and research. Without urgent corrective measures, the world risks allowing mpox to become an entrenched and neglected epidemic with far-reaching public health implications.

## Policy implications

The extension of mpox as a public health emergency represents a critical opportunity to reinvigorate political commitment, secure sustained and predictable financing, and galvanise both regional cooperation and global solidarity in the fight against mpox. At a time when the threat of endemic transmission and viral evolution looms large, national governments, regional bodies, and international partners must rally behind a coordinated response to prevent mpox from becoming a protracted and neglected health crisis. Therefore, reaffirming mpox as a public health emergency enables:

### Mobilisation of political leadership and emergency funding and vaccines

The reclassification of mpox as a public health emergency creates the necessary political urgency to unlock rapid disbursement of emergency funds from domestic budgets, multilateral financing instruments, and donor agencies. It signals to development partners and global health institutions that immediate, predictable, and sustained support is essential to contain the spread, strengthen response systems, and prevent further loss of life. This designation also facilitates the expedited procurement and equitable distribution of critical medical countermeasures, including IPC supplies and vaccines. Importantly, it reinforces the need to not only expand vaccine access but also to invest in and accelerate regional manufacturing capacities across Africa. Strengthening local production of vaccines, diagnostics and therapeutics is a cornerstone of building the continent’s self-reliance and reducing dependency on external supply chains. By linking emergency response with long-term resilience, the mpox emergency declaration becomes a catalyst for affirming Africa’s leadership and independence in addressing current and future health threats.

### Deployment of emergency coordination teams to hotspots

Emergency designation facilitates the rapid activation and deployment of multidisciplinary IMST to mpox hotspots. These teams have proven their strategic value at both continental and national levels by ensuring that outbreak responses are coordinated, standardised, and grounded in evidence-based practices aligned with international protocols. They have also played a key role in promoting more equitable distribution of resources – such as funding and vaccines – while tracking implementation performance and providing timely reporting to regional and global coordination platforms. Building on these successes, it is now critical to decentralise IMSTs further to high-burden provinces and districts. Localised deployment will enhance real-time decision-making, accelerate containment measures, and bring the response closer to affected communities – ultimately increasing impact and responsiveness at the frontlines.

### Strengthening cross-border and community-based surveillance

The designation of mpox as a public health emergency necessitates the full integration of surveillance, reporting, and response activities and reinforces the need for robust cross-border surveillance systems to detect and respond to cases in real-time. In regions characterised by porous borders and high population mobility, cross-border collaboration – including data sharing, synchronised outbreak investigations, and harmonised public health measures – is indispensable for disrupting transmission chains and preventing international spread. Equally vital is the reinforcement of community-based surveillance as the first line of defence. Community health workers, equipped with appropriate training, tools, and support, play a central role in identifying suspected cases, facilitating early referral, and disseminating risk communication. Their trusted presence at the grassroots level enables timely detection and localised response, especially in remote or underserved areas.

### Strengthened genomic surveillance and decentralised laboratory diagnostic capacity

Reaffirming mpox as a public health emergency reinforces the urgency of investing in robust laboratory systems, with a dual focus on expanding genomic surveillance and decentralising diagnostic capacity. Genomic sequencing and bioinformatics are critical for tracking viral evolution, identifying emerging clades, and informing real-time response strategies. However, these efforts must be complemented by strengthening routine diagnostic capacity at subnational levels to ensure timely detection and confirmation of cases closer to where outbreaks occur. Decentralised laboratory infrastructure – including rapid testing capabilities, cold chain systems, and trained personnel – enhances the speed and equity of response, particularly in remote or underserved regions. Strategic investment in genomic and diagnostic capacity enhances the mpox response and builds broader preparedness for future zoonotic and emerging disease threats, aligned with One Health principles.

### Community protection through risk communication, infection prevention and control, and vaccination

Reaffirming mpox as a public health emergency creates the momentum and policy space needed to scale up critical community-level interventions. Among the most immediate and impactful consequences is the ability to strengthen community protection through integrated risk communication, IPC, and equitable vaccination strategies. Risk communication empowers individuals with timely, accurate, and culturally sensitive information to recognise symptoms, adopt preventive behaviours, and combat misinformation. In parallel, reinforcing IPC measures – such as hand hygiene, use of protective equipment, safe caregiving, and environmental sanitation – at both household and health facility levels is essential to interrupt transmission and safeguard frontline workers. Vaccination, particularly for priority groups such as health workers, close contacts of confirmed cases and key populations, remains a central pillar of prevention. Together, these interventions form the foundation of an effective, community-centred response to mpox.

## Conclusion

Mpox remains a serious and underrecognised public health emergency in Africa. Without sustained attention, investment, and coordination, the continent faces the continued risk of rising morbidity, mortality, and potential global resurgence. This joint declaration by Africa CDC and WHO signals a renewed commitment to mpox control and elimination – rooted in science, solidarity, and sovereignty. In a globalised world, health security is indivisible: an injury to one is an injury to all. The unchecked spread of mpox in one region can quickly become a threat to all, as borders offer no protection against emerging pathogens. As such, the protection of Africa’s health systems must be viewed not only as a regional imperative but also as a global priority. The security of one is the security of everyone. Now is the time to act decisively – together – to ensure that mpox does not become a neglected epidemic, but rather a testament to the power of global solidarity and shared responsibility in protecting human health.

## References

[1] Mercy K, Tibebu B, Fallah M, Faria NR, Ndembi N, Tebeje YK. Mpox continues to spread in Africa and threatens global health security. Nat Med. 2024;30(5):1225–1226. 10.1038/s41591-024-02862-6 38472296 PMC11586445

[2] Nachega JB, Sam-Agudu NA, Ogoina D, et al. The surge of mpox in Africa: A call for action. Lancet Glob Health. 2024;12(7):e1086–e1088. 10.1016/S2214-109X(24)00187-6 38735300

[3] Taylor L. WHO and African CDC declare mpox a public health emergency. BMJ. 2024 Aug 16;386:1809. 10.1136/bmj.q1809. 39151943

[4] Lee SS, Traore T, Zumla A. The WHO mpox public health emergency of international concern declaration: Need for reprioritisation of global public health responses to combat the MPXV Clade I epidemic. Int J Infect Dis. 2024;147:107227. 10.1016/j.ijid.2024.107227 39209149

[5] Ndembi N, Folayan MO, Ngongo N, et al. Mpox outbreaks in Africa constitute a public health emergency of continental security. Lancet Glob Health. 2024;12(10):e1577–e1579. 10.1016/S2214-109X(24)00363-2 39178878

[6] Sun J, Zhou L, Wu B, et al. Characteristics of the first confirmed case of human infection with mpox virus clade Ib in China. Nat Commun. 2025;16(1):1–8. 10.1038/s41467-025-61038-z 40425606 PMC12116847

[7] Pareek A, Singhal R, Pareek A, Chuturgoon A, Apostolopoulos V, Chattu VK. Global spread of clade Ib mpox: a growing concern. Lancet Microbe. 2025;101132. 10.1016/j.lanmic.2025.101132 40215986

[8] Beiras CG, Malembi E, Escrig-Sarreta R, et al. Concurrent outbreaks of mpox in Africa – An update. Lancet. 2025;405(10472):86–96. 10.1016/S0140-6736(24)02353-5 39674184

